# Community-based Participatory Research in the Lens of Social Capital: A Mental Health Literacy Promotion Project

**DOI:** 10.1093/geroni/igaf122.142

**Published:** 2025-12-31

**Authors:** Jessie Ho-Yin Yau, Wing-Yin For, Ken Tsz-Kin Ho, Stella Lai-Kuen Wong, Cheryl Hiu-Kwan Chui, Terry Lum

**Affiliations:** The University of Hong Kong, Hong Kong, China (People’s Republic); The University of Hong Kong, Hong Kong, China (People’s Republic); The University of Hong Kong, Hong Kong, China (People’s Republic); The University of Hong Kong, Hong Kong, China (People’s Republic); The University of Hong Kong, Hong Kong, China (People’s Republic); The University of Hong Kong, Hong Kong, China (People’s Republic)

## Abstract

Community-based participatory research (CBPR) is an approach that integrates community knowledge into evidence-based practice, enhancing intervention validity. While existing CBPR models were developed with a top-down approach in the West, their applicability in East Asian contexts and validity may be limited. CBPR principles emphasize resource and information sharing, yet current models overlook the synergy between network and resource sharing that influences collective actions and outcomes. This study aimed to address these gaps by co-creating a conceptual framework with stakeholders to understand CBPR’s operational mechanism from a social capital perspective. The research focused on a mental health literacy promotion project in an East Asian community, involving nine focus group discussions with older adults (n = 21) and social workers (n = 15), followed by co-analyzing and co-interpreting the data and findings with community stakeholders. Our data suggested six critical elements, which can be broadly grouped under the three primary forms of social capital, underlie the process of CBPR: emotional meaningfulness within a group as a foundation for sustainability, characterized by collective harmony, shared values and norms, and role models (bonding social capital); co-learning and co-production of resources across the groups, stimulated by the knowledge exchange environment (bridging social capital); and breaking hierarchical gaps and fostering equal partnership, induced by resource contributions from each stakeholder and the existence of super connectors (linking social capital). These elements promote network, skills, knowledge, and resource sharing among various stakeholders for collective actions for sustainability, community capacity building, and the common good.

